# Optimal dose limitation strategy for bone marrow sparing in intensity-modulated radiotherapy of cervical cancer

**DOI:** 10.1186/s13014-019-1324-y

**Published:** 2019-08-05

**Authors:** Zhirong Bao, Dajiang Wang, Shupeng Chen, Min Chen, Dazhen Jiang, Chunxu Yang, Hui Liu, Jing Dai, Conghua Xie

**Affiliations:** 1 Department of Radiation and Medical Oncology, Hubei Key Laboratory of Tumor Biological Behaviors, Hubei Cancer Clinical Study Center, Zhongnan Hospital of Wuhan University, Wuhan, China; 2 Department of Radiation Oncology, William Beaumont Hospital, 3601 W. 13 Mile Rd, Royal Oak, MI 48073 USA

**Keywords:** Bone marrow sparing, Dose limitation, Cervical cancer, Intensity-modulated radiotherapy

## Abstract

**Background:**

To quantify the dosimetric parameters of different bone marrow sparing strategies and to determine the optimal strategy for cervical cancer patients undergoing postoperative intensity-modulated radiotherapy (IMRT).

**Methods:**

Fifteen patients with cervical cancer were selected for analysis. The planning target volume (PTV) and the organs at risks (OAR) including small bowel, bladder, rectum, femoral heads, os coxae (OC), lumbosacral spine (LS) and bone marrow (BM) were contoured. For each patient, four IMRT plans with different strategies were generated, including one plan without BM as the dose-volume constraint, namely IMRT (N) plan, and three bone marrow sparing (BMS-IMRT) plans. The three BMS-IMRT plans used the BM, OC, OC and LS respectively, as the BM OAR, namely as IMRT (BM), IMRT (OC) and IMRT (OC + LS) plans. Dose volumes for the target and the OARs were compared using analysis of variance (ANOVA).

**Results:**

Compared with IMRT (N) plans, the dose to the small bowel, bladder, rectum and femoral heads showed no increase in the three BMS-IMRT plans. However, the irradiated dose to BM, OC and LS significantly decreased. In particular, the mean dose of BM, OC and LS decreased by about 5Gy (*p* < 0.05) in IMRT (BM) plans while the average volume receiving ≥20, ≥30, ≥40Gy decreased by 7.1–24.2%. The LS volume receiving 40Gy showed the highest decrease (about 31.2%, *p* < 0.05) in IMRT (OC + LS) plans. On the other hand, in comparison with IMRT (BM), IMRT (OC) reduced the dose volume of to the OC, but increased the dose to LS while IMRT (OC + LS) plans reduced both the OC and the LS volume at all dose levels. Specifically, the V_20_ of OC and LS in the IMRT (OC + LS) plan decreased by 11.5 and 11.2%, respectively.

**Conclusion:**

By introducing the os coxae and lumbosacral spine as the dose–volume constraints, the IMRT plans exhibited the best sparing of the bone marrow without compromising the dose to surrounding normal structures. Therefore, we recommend adding the os coxae and lumbosacral spine as the BM OAR in such plans.

## Background

Adjuvant pelvic radiotherapy with concurrent chemotherapy is the standard treatment approach or cervical cancer patients who have previously undergone hysterectomy [1]. However, the use of chemoradiotherapy increases the risk of developing serious hematologic toxicity (HT), which can impair the delivery of chemotherapy and may result in treatment interruptions [2, 3]. Thus, the reduction of HT is crucial.

Studies have shown the advantages of pelvic intensity-modulated radiation therapy (IMRT), including better dosimetric distribution, relatively lower irradiation dose to normal tissues and fewer acute side effects, compared with conventional forward planning techniques [4, 5]. However, the exposure of bone marrow (BM), especially of the ilium and lumbosacral spine, remains unavoidable. During pelvic IMRT, a large volume of BM is irradiated, along with other critical normal tissues such as the small bowel, bladder, rectum and femoral heads, and hence the irradiation is unavoidable. Due to the high radiosensitivity of BM, radiation can induce acute and chronic pathologic and radiographic changes to the BM and lower BM activity [6, 7]. Therefore, an effective planning strategy to limit bone marrow irradiation and limit the incidence of HT is needed.

Studies have demonstrated the occurrence of acute HT is associated with the volume of BM irradiated, especially the volume of lumbosacral spine (LS) and lower pelvis irradiated [8–10]. Thus, a reduction of the radiation dose to the LS of the BM could be particularly beneficial. In the recent bone marrow sparing IMRT (BMS-IMRT), the experience with BMS-IMRT is limited [11–13]. In general, the whole BM or the iliac is used as the avoidance structure for IMRT planning. Other subsites of BM, such as the lumbosacral spine, ischium and pubis, where most HT occurs [8–10, 14], were not defined. Optimizing IMRT plans to focus on avoiding iliac alone might shift the dose to other regions, while sparing the entire BM may adversely affect the sparing of other organs at risks (OAR). Therefore, we proposed that the BM sparing plans should focus not only on avoiding the iliac crests but also on other BM subsites. We hypothesized that if the os coxae (OC) and/or LS were defined as separate OAR intentionally, it might be possible to decrease the dose to the bone marrow, while keeping the dose to other OAR at an acceptable level. In the present study, we investigated the bone marrow sparing using different dose limitation strategies and determined the optimal strategy for treating patients with cervical cancer.

## Methods

### Patient selection and simulation

Fifteen cervical cancer patients treated with pelvic IMRT in our institution between January 2018 and July 2018 were selected for this study. Inclusion criteria were (1) staged IB–IIB, (2) biopsy-proven squamous cell carcinoma and (3) undergoing postoperative pelvic radiation therapy. Patients with high-risk pelvic lymph nodes receiving simultaneous integrated boost were excluded. The mean and median age of eligible patients was 56.3 and 61 years (range, 42–65), respectively.

All patients underwent CT simulation on a helical CT scanner (Sensation Cardiac 64x, Siemens, Munich, Bavaria, Germany) with 3 mm slice thickness. The scans were collected from the L1 vertebra to the region of 5 cm below the ischial tuberosities. Patients were immobilized with a vacuum-formed cradle in the supine position with comfortably full bladder and no bowel preparation prior to simulation.

### Normal tissue definition

**Fig. 1:**
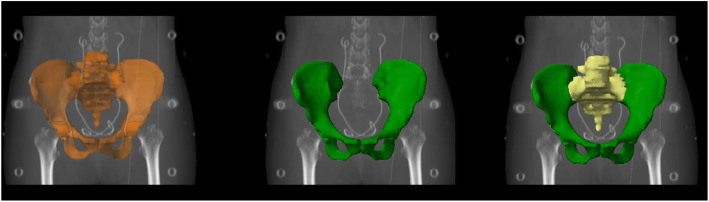
Coronal section illustrating delineation of os coxae (green), lumbosacral spine (yellow) and bone marrow (brown)

**Fig. 2:**
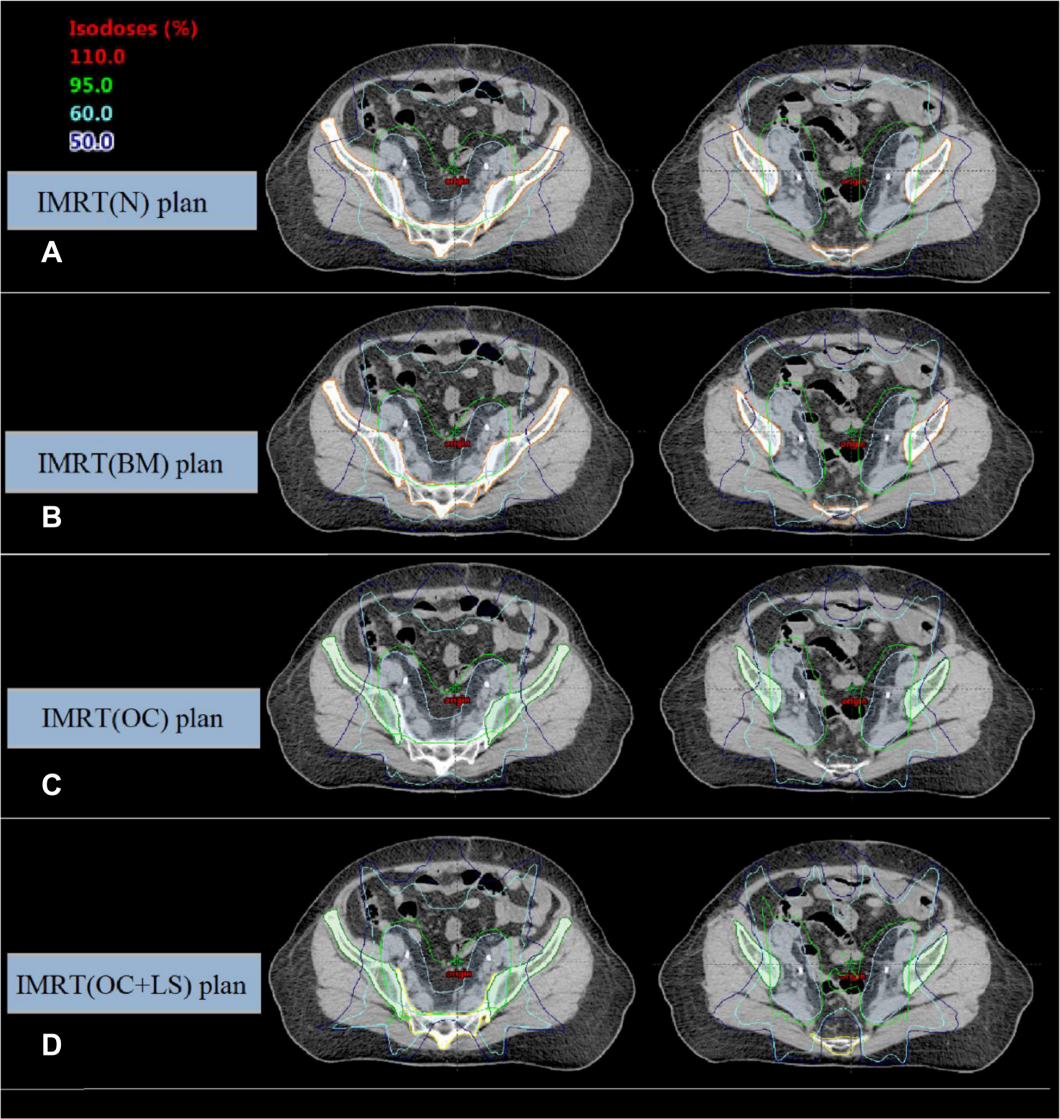
Axial dose distributions obtained by **a**: IMRT (N), **b**: IMRT (BM), **c**: IMRT (OC) and **d**: IMRT (OC + LS) plans. Structures included planning target volume (blue), os coxae (green), lumbosacral spine (yellow) and bone marrow (brown)

Normal tissue included small bowel, bladder, rectum, femoral heads and bone marrow (BM). For each patient, the small bowel was contoured consisted of as the entire peritoneal cavity from L4-L5. The external contour of bone marrow was delineated (Figs. 1 and 2), rather than the low-density regions within the bones, to ensure reproducibility and minimize dependence of the contours on CT windowing and leveling. The entire BM was divided into two subsites: (1) os coxae (OC)—defined as the region extending from the iliac crests to the ischial tuberosities comprising the ilium, pubis, ischium and acetabula but not including the femoral heads; (2) lumbosacral spine (LS)—extending from the superior border of the L5 vertebra to the coccyx. The OC, LS and BM were contoured separately for planning constraints.

### Target definition and treatment planning

For consistency, all contours were delineated by a single radiation oncologist. The clinical target volume (CTV) encompassed the gross tumor volume and potentially microscopic disease, extending from the L4-L5 vertebra to the inferior border of the obturator foramen. In detail, the CTV was generally comprised of the upper vagina, parametrial tissues, uterus (if present), and regional lymph nodes (common iliac, external and internal iliac, obturator, and presacral nodes). Considering organ motion and setup error, a uniform 7 mm margin was applied to the CTV to generate the planning treatment volume (PTV). The 7 mm margin was determined using the van Herk methodology [15] with the measurements of systematic setup errors and individual random errors obtained from the patients of our institution.

For consistency, all the treatment planning procedures were developed by the same radiation physicist. The inverse planned dynamic IMRT plans were prepared using Eclipse treatment planning system version 13.5 (Varian Medical Systems, Palo Alto, CA, USA). The analytic anisotropic algorithm (AAA) with the grid size of 2.5 mm was used for computing dose to the irradiated region. The machine parameter optimization method was used with 6 MV photon beams, sliding-window fields and a multileaf collimator leaf size of 5 mm. All plans in the study were designed to be executed using Varian iX linear accelerator (Varian Medical Systems, Palo Alto, CA, USA) at a dose rate of 400 MU/min, with cone beam computed tomography (CBCT) scanners on-board for daily image guidance before treatment. The CBCT images were acquired with scan parameters 125 kV, 80 mA and 3 mm slice thickness in the half-fan mode using a half bowtie filter. An automatic rigid registration process of the CBCT to the planning CT was performed in three translational directions, including left–right, superior–inferior, and anterior–posterior. To improve the set-up accuracy, the trained radiation therapist first evaluated the automatic bony match in the pelvis clip box and made necessary manual adjustments to acquire satisfactory bony matching. Then, the radiation oncologist checked whether the CTV was covered by the PTV. When deviations exceeded 5 mm, we would considered repositioning the patient, performing CBCT again and repeating the aforementioned matching process. Generally, the first CBCT procedure was verified by the radiation oncologist online while subsequently ones were verified via the offline review.

For each patient, four inverse IMRT plans were generated: IMRT (N) plan without BM as the sparing objective, IMRT (BM) plan using total BM as the sparing objective, IMRT (OC) plan using OC as the sparing objective and IMRT (OC + LS) plan using OC and LS as the sparing objective. The plans were optimized with equally spaced nine coplanar fields, with gantry angles of 180, 140, 100, 60, 20, 340, 300, 260 and 220. A dose of 50.4 Gy in 28 fractions was delivered to the PTV.

In the optimization process, all plans were generated adopting an identical set of PTV/OAR dose–volume constraints to keep the results comparable. The optimization parameters are similar to the RTOG 0418 [16]. In all four IMRT plans, the PTV was given the highest priority. The criterion for acceptance of the plan was that at least 95% of the PTV received 100% of the prescription dose, and with the maximal dose in the PTV<110% of the prescription dose. The priority was set higher for the small bowel, rectum, BM, OC and LS relative to the femoral heads and bladder, accounting for the importance. The OAR volumes were used directly instead of the ring volumes. Details about the dose constraints and weightings are summarized in Table 1. Optimization proceeded with these settings until no further improvement occurred. Then, the field fluences were converted to the leaf motion of dynamic multileaf collimator, and the dose distribution was calculated. In general, the plans should be optimized in an iterative fashion for a second time after one optimization was performed for the first time to obtain the optimal dose distribution. The optimizations of BMS-IMRT plans started from scratch, instead of starting from plans that had already been optimized for the other OAR.

**Table 1:** The dose-volume constraints

Structure	Dose-volume constraints	Relative Priority
PTVo	D_100%_ ≥ 50.70 Gy	150
	D_max_ ≤ 53.50 Gy	150
PTVi	D_100%_ ≥ 50.70 Gy	150
	D_max_ ≤ 51.50 Gy	150
BM	D_mean_ ≤ 32.00 Gy	80
OC	D_mean_ ≤ 28.00 Gy	80
LS	D_mean_ ≤ 35.00 Gy	80
Small bowel	D_max_ ≤ 52.50 Gy	80
	V_40_ < 30%	80
Rectum	D_max_ ≤ 52.50 Gy	80
	V_40_ < 50%	80
Bladder	V_45_ < 35%	50
Femoral head (left)	V_50_ < 2%	50
Femoral head (right)	V_50_ < 2%	50
Body	D_max_ ≤ 53.50 Gy	300

*Abbreviations*: *PTVo* The planning target volume excluding the small bowel, *PTVi* The planning target volume inside the small bowel, *D*_*max*_ The maximum dose received, *D*_*mean*_ The mean dose, *D*_*n%*_ Dose received by the n% volume of the target volume, *V*_*x*_ Percentage volume irradiated by *x* Gy or more of a certain structure

### Plan comparison

Data from the dose volume histograms (DVHs) acquired for all contoured organs and the target volume was analyzed. For the PTV, dosimetric parameters were quantified, including the mean dose (D_mean_), the minimum point dose (D_min_), the maximal point dose (D_max_), D_98%_, D_50%_, D_2%_, conformity index (CI) and homogeneity index (HI).

CI was used to assess the conformity of dose distribution.

$$ \mathrm{CI}=\frac{V_{t, ref}}{V_t}\times \frac{V_{t, ref}}{V_{ref}} $$

Here, *V*_*t,ref*_ was the target volume receiving the prescribed dose, *V*_*t*_ was the target volume and *V*_*ref*_ was the total volume covered by the prescribed dose. A value of CI close to 1 reflects an improved PTV conformity.

According to ICRU repot NO.83 [17], HI is suggested as follows:

$$ \mathrm{HI}=\frac{D_{2\%}-{D}_{98\%}}{D_{50\%}} $$

HI is defined to assess the homogeneity of dose distribution. Here, D_2%_ represented the dose received by 2% volume of the target, D_98%_ was the dose received by 98% volume of the target and D_50%_ was the median absorbed dose. A value of HI close to 0 means an ideal uniform dose.

For the OAR (small bowel, bladder, rectum, femoral heads, BM, OC and LS), a set of dosimetric parameters was obtained, including the mean dose (D_mean_), the V_10_ (the percent of volume that received 10Gy), V_20_, V_30_, V_40_ and V_50_.

### Data analysis

Once the treatment planning was completed, the plan was normalized to cover 95% of the PTV with the prescribed dose to keep the results comparable. Then the DVH parameters of PTV and OAR were analyzed using SPSS (Version 20.0; SPSS, Inc., Chicago, IL, USA). Analysis of variance (ANOVA) was used to test the differences of the DVH parameters obtained from different planning strategies and *p* < 0.05 was considered to be statistically significant.

## Results

### PTV coverage

The PTV volume was 1061 ± 110 cm^3^ (range, 875–1224). The PTV planning objectives were achieved with four different dose limitation strategies and all plans were normalized to cover 95% of the PTV with ≥100% of the prescribed dose. Table 2 shows the dosimetric parameters for PTV.

**Table 2:** Dosimetric parameters for PTV

Parameters	IMRT (N)	IMRT (BM)	IMRT (OC)	IMRT (OC + LS)
D_min_ (Gy)	46.13 ± 1.79	44.28 ± 1.80*	43.77 ± 2.23*	42.70 ± 2.03*^†^
D_max_ (Gy)	53.78 ± 0.28	54.25 ± 0.36*	54.45 ± 0.38*	54.92 ± 0.40*†
D_mean_ (Gy)	51.59 ± 0.10	51.68 ± 0.07*	51.65 ± 0.07*	51.71 ± 0.09*
CI	0.87 ± 0.02	0.89 ± 0.01*	0.88 ± 0.02^†^	0.87 ± 0.02^†^
HI	0.06 ± 0.00	0.06 ± 0.00*	0.06 ± 0.00*	0.07 ± 0.00*†

*Abbreviations*: *HI* Homogeneity index, *CI* Conformity index, *D*_*min*_ the minimum dose, *D*_*max*_ the maximum dose, *D*_*mean*_ the mean dose, *IMRT (N)* without bone marrow as avoidance structures, *IMRT (BM)* whole bone marrow as avoidance structures, *IMRT (OC)* os coxae as avoidance structures, *IMRT (OC + LS)* both os coxae and lumbosacral spine as avoidance structures

*p value < 0.05 while comparing IMRT (N) plan with other three plans

^†^*p* value < 0.05 while comparing IMRT (BM) plan with IMRT (OC) and IMRT (OC + LS) plans, respectively

The dose distribution in the PTV satisfied the clinical requirement of less than 2% of the PTV receiving more than 107% of the prescribed dose. As shown in Table 2, compared with IMRT (N), the maximal dose increased by 0.9–2.1% (*p* < 0.05) while the minimal dose decreased by 4.0–7.4%(*p* < 0.05) for the BMS-IMRT plans. Compared with IMRT (BM), the maximal PTV dose increased and the minimal dose decreased significantly in the IMRT (OC), and IMRT (OC + LS) plans, with the differences within 3.7%. In terms of CI and HI, IMRT (BM) plan and IMRT (N) plan showed the slightly better conformity and homogeneity than did in IMRT (OC + LS) plans, but they resulted in a greater dose to the bone marrow. Typical dose distributions for the four strategies compared in this study are shown in Fig. 2.

### Dose distribution for bowel, rectum, bladder, and femoral heads

Table 3 listed the volumes of small bowel, rectum, bladder and femoral heads, respectively, receiving ≥10, ≥20, ≥30, ≥40 and ≥ 50Gy. In general, IMRT (BM), IMRT (OC), and IMRT (OC + LS) plans reduced the irradiated volume of the rectum, small bowel, bladder and femoral heads.

**Table 3:** Dosimetric parameters for small bowel, rectum, bladder and femoral heads

Volume	IMRT (N)	IMRT (BM)	IMRT (OC)	IMRT (OC + LS)
Small Bowel				
V_10_ (%)	84.83 ± 11.28	79.19 ± 24.78	78.69 ± 24.67	78.75 ± 24.69
V_20_ (%)	72.19 ± 11.65	66.44 ± 21.92	68.90 ± 22.21	65.29 ± 21.72
V_30_ (%)	50.96 ± 12.88	49.13 ± 18.04	51.03 ± 17.64	47.93 ± 17.64
V_40_ (%)	28.30 ± 10.79	26.44 ± 12.75	27.65 ± 12.41	26.38 ± 12.09
V_50_ (%)	7.87 ± 4.13	7.80 ± 4.79	7.96 ± 5.01	8.53 ± 4.99
Rectum				
V_10_ (%)	96.80 ± 4.96	96.72 ± 5.23	96.69 ± 5.25	96.70 ± 5.22
V_20_ (%)	90.29 ± 5.69	89.30 ± 5.94	90.06 ± 5.93	89.45 ± 6.14
V_30_ (%)	74.09 ± 5.92	74.30 ± 4.90	74.36 ± 4.71	73.72 ± 3.84
V_40_ (%)	53.89 ± 7.45	54.45 ± 6.40	54.57 ± 6.77	54.60 ± 4.36
V_50_ (%)	25.27 ± 8.17	26.07 ± 8.26	26.01 ± 8.32	27.26 ± 7.70
Bladder				
V_30_ (%)	95.40 ± 4.14	92.25 ± 4.70	91.00 ± 5.59	88.57 ± 5.97
V_40_ (%)	72.03 ± 5.43	66.47 ± 10.30	63.63 ± 11.58	61.53 ± 11.81
V_50_ (%)	29.49 ± 13.27	29.39 ± 13.96	29.45 ± 13.48	30.05 ± 13.94
Femoral head (left)				
V_30_ (%)	15.63 ± 4.29	10.03 ± 3.94*	8.37 ± 3.69	7.71 ± 3.78
V_40_ (%)	5.98 ± 3.35	2.86 ± 2.60*	2.51 ± 2.52	2.39 ± 2.48
V_50_ (%)	0.46 ± 0.75	0.34 ± 0.70	0.38 ± 0.72	0.39 ± 0.69
Femoral head (right)				
V_30_ (%)	14.96 ± 4.15	9.19 ± 3.29*	7.39 ± 2.97	7.20 ± 3.37
V_40_ (%)	5.20 ± 2.90	2.43 ± 2.08*	2.01 ± 2.00	1.98 ± 1.99
V_50_ (%)	0.34 ± 0.62	0.20 ± 0.43	0.30 ± 0.59	0.32 ± 0.59

*Abbreviations*: *IMRT (N)* without bone marrow as avoidance structures, *IMRT (BM)* whole bone marrow as avoidance structures, *IMRT (OC)* os coxae as avoidance structures, *IMRT (OC + LS)* Both os coxae and lumbosacral spine as avoidance structures, *V*_*n*_ percentage of volume receiving *n* Gy

*p value < 0.05 while comparing IMRT (N) plan with other three plans

^†^*p* value < 0.05 while comparing IMRT (BM) plan with IMRT (OC) and IMRT (OC + LS) plans, respectively

No significant differences among the four strategies for the dosimetric parameters of small bowel, rectum and bladder, meaning that any marrow sparing is not at the expense of other OAR sparing. Additionally, compared with the IMRT (N) plan, the V_30_ and V_40_ of femoral heads showed a decrease in the IMRT (BM) plan (left: 5.6 and 3.1%, right: 5.6 and 3.1%, *p* < 0.05), which may be caused by the dose limitation of the adjacent pelvis bone marrow. Briefly, the three bone marrow-sparing plans showed no significant dose reductions to the small bowel, bladder and rectal dose compared with conventional planning methods. Furthermore, comparing these parameters for the IMRT (BM) plans with the IMRT (OC) and IMRT (OC + LS) plans, respectively, we found no significant differences.

### Dose distribution for BM, OC and LS

**Fig. 3:**
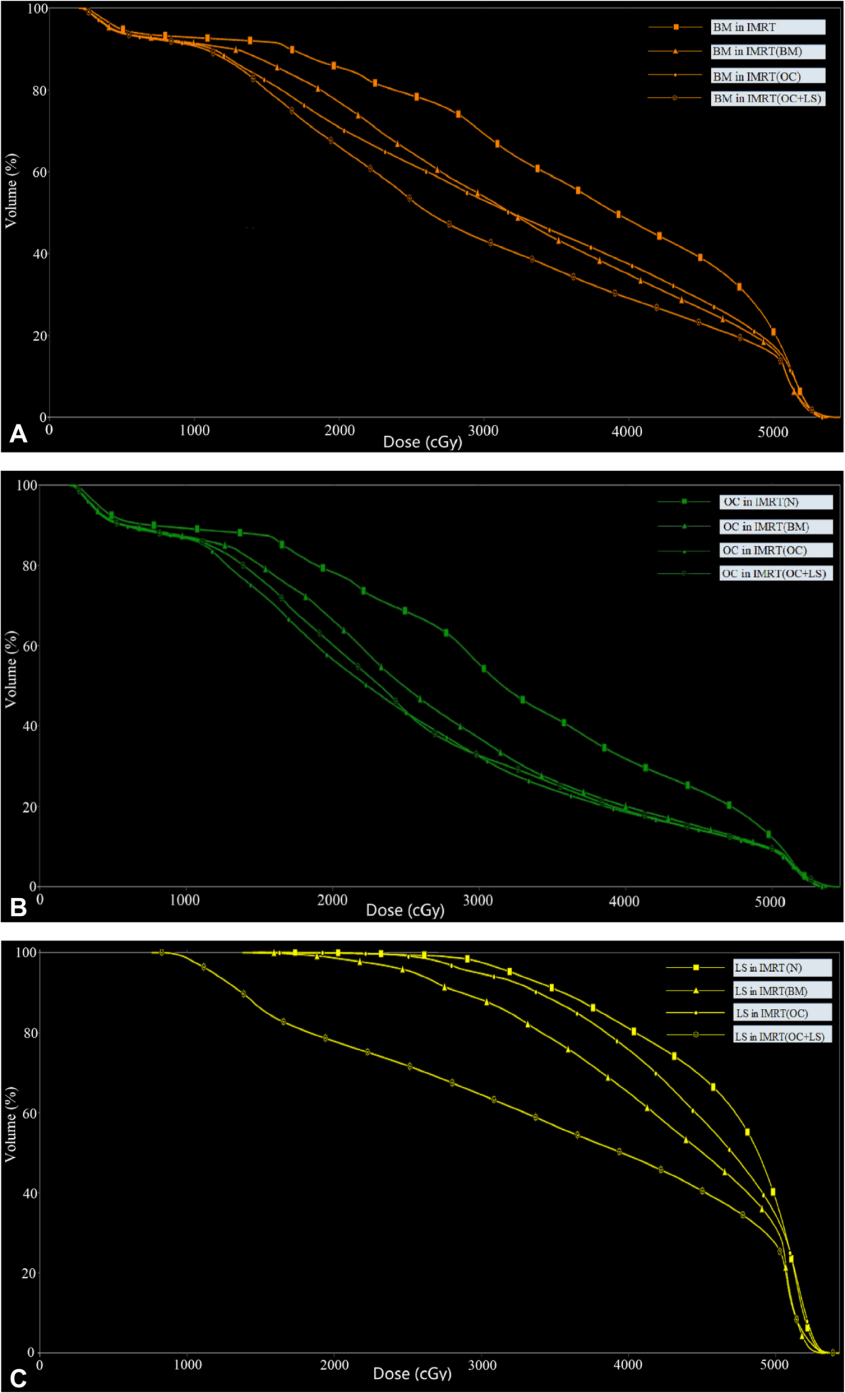
Dose–volume histograms for pelvic bone marrow with four different dose limitation strategies for pelvic radiotherapy. Structures included **a**: BM (brown), **b**: OC (green), and **c**: LS (yellow)

Table 4 summarizes the average dosimetric parameters of the BM, OC and LS and the results of the pairwise statistical analysis. The typical dose-volume histograms for BM, OC and LS are shown in Fig. 3. All the BMS-IMRT plans were superior to the IMRT (N) plans in reducing the mean dose and the volume of BM, OC and LS at all dose levels, except for the LS volume receiving 10 Gy in IMRT (BM) and IMRT (OC).

**Table 4:** Dosimetric comparison for BM, OC and LS

Parameters	IMRT (N)	IMRT (BM)	IMRT (OC)	IMRT (OC + LS)
BM				
D_mean_ (Gy)	37.57 ± 1.11	32.14 ± 0.25*	32.07 ± 0.61*	29.40 ± 0.52*†
V_10_ (%)	97.62 ± 1.98	95.46 ± 2.04*	92.72 ± 2.51*†	92.74 ± 2.31*†
V_20_ (%)	88.38 ± 2.87	76.88 ± 1.99*	70.58 ± 2.00*†	65.67 ± 1.44*†
V_30_ (%)	72.17 ± 3.67	53.70 ± 1.54*	55.41 ± 2.20*	45.25 ± 1.50*†
V_40_ (%)	50.03 ± 4.07	33.47 ± 1.70*	38.64 ± 2.83*†	29.63 ± 1.90*†
V_50_ (%)	20.29 ± 2.42	15.79 ± 1.64*	17.11 ± 1.83*	14.77 ± 1.47*
OC				
D_mean_ (Gy)	33.46 ± 1.46	28.27 ± 0.93*	25.42 ± 0.46*†	25.78 ± 0.59*†
V_10_ (%)	96.21 ± 3.15	92.94 ± 3.05*	88.47 ± 3.67*†	89.06 ± 3.31*†
V_20_ (%)	81.97 ± 4.24	67.99 ± 2.74*	53.81 ± 1.84*†	56.51 ± 1.59*†
V_30_ (%)	58.72 ± 4.66	40.41 ± 2.12*	33.34 ± 1.49*†	33.82 ± 1.56*†
V_40_ (%)	35.01 ± 3.76	23.00 ± 2.44*	20.60 ± 1.93*†	20.75 ± 2.22*†
V_50_ (%)	14.11 ± 2.42	11.22 ± 1.86*	10.69 ± 1.75*	10.63 ± 1.74*
LSBM				
D_mean_ (Gy)	44.55 ± 1.54	38.81 ± 1.98*	43.37 ± 1.48*	35.51 ± 0.30*
V_10_ (%)	99.98 ± 0.07	99.77 ± 0.69	99.98 ± 0.07	99.05 ± 1.47*†
V_20_ (%)	99.21 ± 1.52	92.14 ± 5.07*	98.86 ± 1.48†	80.97 ± 2.51*†
V_30_ (%)	94.99 ± 3.18	76.59 ± 7.81*	92.79 ± 3.35†	64.46 ± 1.33*†
V_40_ (%)	75.70 ± 8.58	51.47 ± 6.89*	69.51 ± 8.60*†	44.54 ± 3.06*†
V_50_ (%)	30.90 ± 5.27	23.63 ± 3.88*	28.12 ± 4.34*†	21.74 ± 2.71*

*Abbreviations*: *IMRT (N)* Without bone marrow as avoidance structures, *IMRT (BM)* Whole bone marrow as avoidance structures, *IMRT (OC)* Os coxae as avoidance structures, *IMRT (OC + LS)* Both os coxae and lumbosacral spine as avoidance structures, *BM* Bone marrow, *OC* Os coxae, *LS* Lumbosacral spine, *D*_*mean*_ the mean dose, *V*_*n*_ percentage of volume receiving *n* Gy

*p value < 0.05 while comparing IMRT (N) plan with other three plans

^†^*p* value < 0.05 while comparing IMRT (BM) plan with IMRT (OC) and IMRT (OC + LS) plans, respectively

**Fig. 4:**
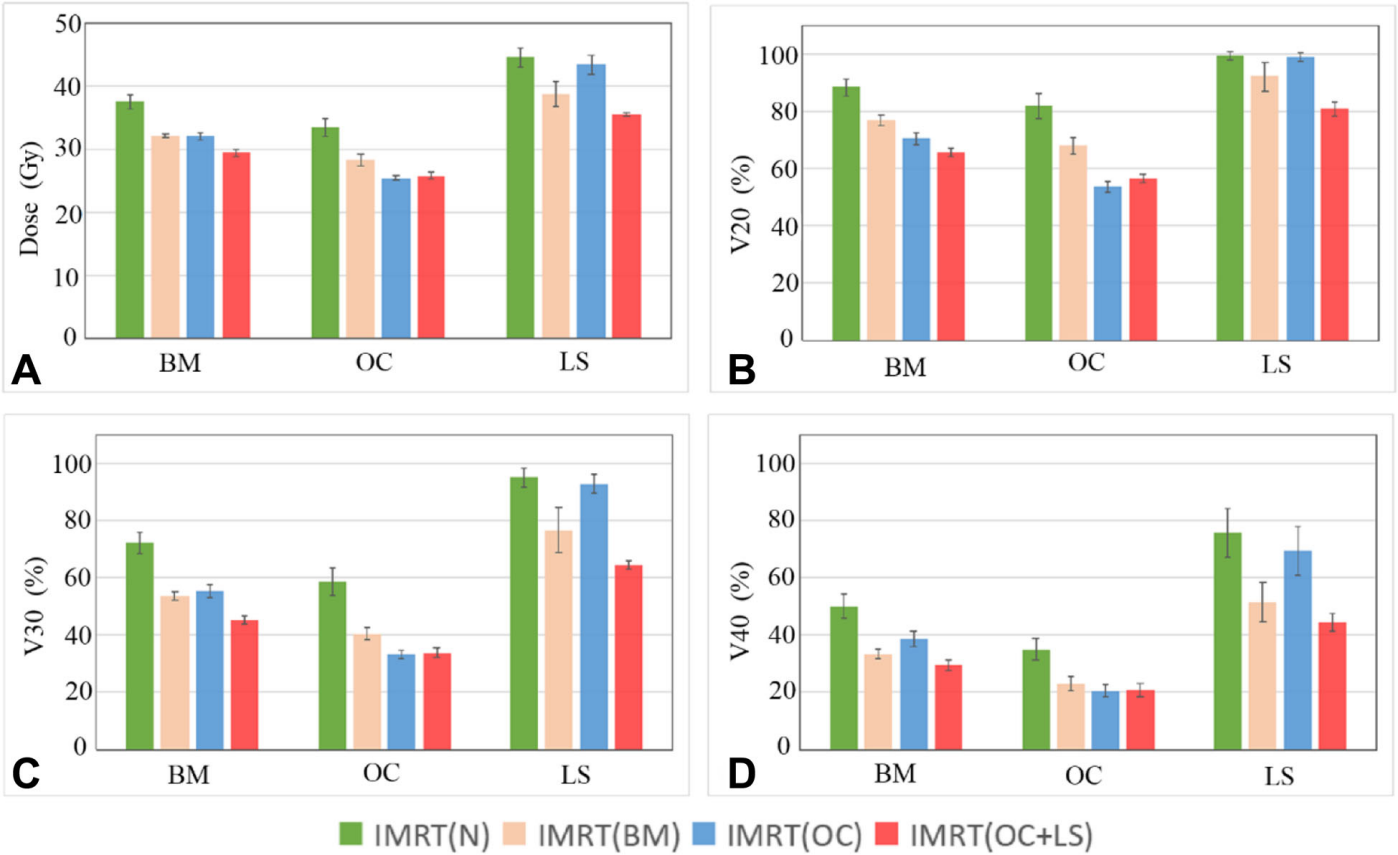
Evaluated mean dose, V_20_, V_30_ and V_40_ for BM, OC and LS in four different strategies. **a**: the mean dose; **b**: V_20_; **c**: V_30_; **d**: V_40_

Compared with the IMRT (N) plans, the mean dose of BM, OC and LS decreased by about 5Gy (*p* < 0.05) in IMRT (BM) plans. As seen in Table 4, the average BM volume receiving ≥20, ≥30, ≥40 Gy decreased by 11.5, 18.5 and 16.6% for IMRT (BM) plans (*p* < 0.05 for each pairwise comparison with IMRT (N) plans). The average V_20_, V_30_ and V_40_ of OC reduced by 14.0, 18.3 and 12.0% while those of the lumbosacral spine reduced by 7.1, 18.4 and 24.2%, respectively (*p* < 0.05) in IMRT (BM) comparison with IMRT (N) plans. The changes in D_mean_, V_20_, V_30_ and V_40_ in the BM, OC and LS were shown in Fig. 4. We found that the radiation dose to bone marrow in IMRT (OC + LS) exhibited a relatively higher reduction than those in other plans. Compared with the IMRT (N), the mean dose to BM, OC and LS decreased by 8.2Gy, 7.7Gy and 9.0Gy in IMRT (OC + LS) while the average LS volume receiving 40Gy showed the highest decrease (about 31.2%, *p* < 0.05).

On the other hand, compared with IMRT (BM) plans, IMRT (OC) reduced the dose volume of OC at different levels, but increased the dose to LS. For this reason, the mean dose to BM were barely lower than that in IMRT (BM) plans (Table 4). In contrast, IMRT (OC + LS) plans reduced both the OC and the LS volume at all dose levels in comparison with IMRT (BM). Specifically, the V_20_ of OC and LS in IMRT (OC + LS) plan decreased by 11.5 and 11.2% compared to IMRT (BM) plan. However, the LSS received a higher radiation dose than the OC, most likely because of its proximity to the PTV.

## Discussion

The present study quantified the dosimetric parameters for different bone marrow sparing strategies by limiting the dose delivered to bone marrow and determined the optimal strategy for cervical cancer patients undergoing postoperative radiotherapy.

Results of this study indicated that our proposed strategy, i.e. adding the os coxae and lumbosacral spine as the planning constraints better spared the bone marrow from excessive radiation without increasing the dose on other normal tissues. In comparison with conventional bone marrow sparing IMRT schemes [18], the proposed strategy enabled both the high-dose and low-dose volume of BM, OC and LS to be decreased, without at the expense of increasing the dose to small bowel, rectum, bladder and femoral heads. Because the definition of the OC and LS is relatively straightforward and not too time consuming, we suggest that the OC and LS should be introduced as the independent OAR to be used for optimizing BMS-IMRT plans.

Although IMRT (OC + LS) plans reduced bone marrow radiation dose while maintaining adequate target volume coverage, the conformity and homogeneity of PTV might be comprised slightly. This may be attributed to the following reason. In these IMRT plans, the treatment load was dispersed in nine evenly spaced fields to satisfy the coverage of the PTV and reduce normal tissue irradiation. For patients with cervical cancer, most of the BM were located in the beam’s eye view [6]. Thus, the additional dose limitation of OC and LS presumably limited the use of degrees of freedom. In this case, IMRT (OC + LS) plans provided inferior dose uniformity and homogeneity of the target compared to the other three methods.

More attention has been devoted in recent years to assess the dose–volume relationship between the amount of pelvic bone irradiated at different dose levels and the risk of acute hematologic toxicity (HT). Mell et al. [9] found the fraction of pelvic bone receiving more than 10Gy was associated with the incidence of Grade 2 or worse leukopenia and neutropenia while Rose [19] and Albuquerque [20] found that patients with increased BM V_20_ were more likely to experience leukopenia. In particular, these similar findings (i.e. V_10_-V_20_ as best predictors) highlighted the strong sensitivity of bone marrow stem cells to radiation at low doses. Other groups, on the contrary, reported that the volume of pelvic BM receiving high-dose radiation (i.e. V_30_, V_40_) significantly correlated with HT [21, 22]. For instance, Bazan et al. [22] found that patients with a mean BM dose of ≥30Gy have a 6.9-fold increase in the odds of developing HT3+ compared to patients with a mean BM dose of ≤30Gy. Although further explorations about the dose–volume relationships are still needed, these studies show that the volume of pelvic BM receiving a certain radiation dose could be a significant contributor of acute HT. Therefore, employing new bone marrow sparing strategies to reduce BM irradiation is necessary.

To the best of our knowledge, the present work is the first to take into account the separate use of the OC and LS for IMRT plans optimization. In the comparative dosimetric study, some DVH statistical parameters of BM, OC and LS decreased significantly. Although possibly beneficial, several important points remain to be addressed.

Firstly, the results showed that the dose delivered to the LS was significantly greater owing to its proximity to the PTV. Excessive dose constraints, even with the optimal BM sparing strategy, may adversely affect the sparing of other normal tissues or compromise the PTV coverage. In this case, other advanced techniques such as Tomotherapy [23] and VMAT [24], due to its higher degrees of freedom, could theoretically result in further sparing of both OC and LS. Further research to evaluate the potential benefits of BM sparing from other techniques is therefore needed. Moreover, the application of highly conformal IMRT techniques as a tool to spare bone marrow also addresses the need for real-time dose verification to improve patient setup reproducibility and reduce the potential difference between planned and delivered dose. Furthermore, daily image guidance would hopefully reduce the planning margins that could limit the irradiation of bone marrow as well.

Another challenge is that the entire bone anatomy is generally contoured and entered as dose–volume constraints in the standard IMRT plans, which overestimates the volume of active BM and constrains optimization. It is known, however, that BM is comprised of both active “red” marrow and inactive “yellow” marrow, which cannot be well visualized on computed tomography (CT) [21] and only the small portion of active BM should be spared preferentially and sufficiently. Functional imaging techniques, such as magnetic resonance imaging (MRI) [25], single photon emission CT (SPECT) [26], and positron emission tomography (PET) [27–29], are potential methods to identify the active BM, in order to use these subregions in IMRT planning as avoidance structures to further reduce BM irradiation. Despite its potential effectiveness, additional investigations to explore the functional bone marrow sparing IMRT are necessary. Moreover, functional imaging is expensive and not universally available.

Finally, the proposed strategy of plan optimization using the OC and LS instead of the whole BM has been found to be more effective in limiting the bone marrow irradiation, compared with the conventional BMS-IMRT scheme. However, it is still unclear to what extent the clinically meaningful reductions in HT could be achieved. Future work should focus on the evaluating the optimization strategy by long term follow-up.

## Conclusions

For patients suffering from cervical cancer, IMRT plans with the os coxae and lumbosacral spine as the dose–volume constraints exhibited the optimal sparing of the bone marrow without increasing the dose to other normal tissues. Such novel dose limitation strategy would be expected to be a promising treatment approach to reduce acute HT.

## Data Availability

Please contact author for data requests.

## Abbreviations

ANOVA: Analysis of variance
BMS-IMRT: Bone marrow sparing intensity modulated radiotherapy
CI: Conformity index
CT: Computed tomography
CTV: Clinical target volume
D_max_: The maximum point dose received
D_mean_: The mean dose
D_min_: The minimum point dose
D_n%_: Dose received by the n% volume of the target volume
HI: Homogeneity index
HT: Hematologic toxicity; BM: bone marrow
IMRT: Intensity modulated radiotherapy
LS: Lumbosacral spine
MRI: Magnetic resonance imaging
OAR: Organs at risk; DVHs: dose volume histograms
OC: Os coxae
PET: Positron emission tomography
PTV: Planning target volume
PTVi: The planning target volume inside the small bowel
PTVo: The planning target volume excluding the small bowel
SPECT: Single photon emission computed tomography
V_x_: Percentage volume irradiated by *x* Gy or more of a certain structure
